# An immune-suppressing protein in human endogenous retroviruses

**DOI:** 10.1093/bioadv/vbad013

**Published:** 2023-02-02

**Authors:** Huan Zhang, Shengliang Ni, Martin C Frith

**Affiliations:** Department of Computational Biology and Medical Sciences, Graduate School of Frontier Sciences, University of Tokyo, Chiba 277-8568, Japan; Department of Computational Biology and Medical Sciences, Graduate School of Frontier Sciences, University of Tokyo, Chiba 277-8568, Japan; Department of Computational Biology and Medical Sciences, Graduate School of Frontier Sciences, University of Tokyo, Chiba 277-8568, Japan; Artificial Intelligence Research Center, AIST, Tokyo 135-0064, Japan; CBBD-OIL, AIST, Tokyo 169-8555, Japan

## Abstract

**Motivation:**

Retroviruses are important contributors to disease and evolution in vertebrates. Sometimes, retrovirus DNA is heritably inserted in a vertebrate genome: an endogenous retrovirus (ERV). Vertebrate genomes have many such virus-derived fragments, usually with mutations disabling their original functions.

**Results:**

Some primate ERVs appear to encode an overlooked protein. This protein is homologous to protein MC132 from Molluscum contagiosum virus, which is a human poxvirus, not a retrovirus. MC132 suppresses the immune system by targeting NF-κB, and it had no known homologs until now. The ERV homologs of MC132 in the human genome are mostly disrupted by mutations, but there is an intact copy on chromosome 4. We found homologs of MC132 in ERVs of apes, monkeys and bushbaby, but not tarsiers, lemurs or non-primates. This suggests that some primate retroviruses had, or have, an extra immune-suppressing protein, which underwent horizontal genetic transfer between unrelated viruses.

**Contact:**

mcfrith@edu.k.u-tokyo.ac.jp

## 1 Introduction

Retroviruses cause significant disease, such as acquired immune deficiency syndrome and adult T-cell leukemia. They have RNA genomes, which undergo reverse transcription into DNA, which is inserted into the host cell’s genome. Occasionally, they infect germ-line cells, in which case the insertion may be inherited by future generations of the host organism: this is termed an endogenous retrovirus (ERV). Vertebrate genomes contain many retrovirus-derived fragments, for example they comprise ∼8% of the human genome. Probably most ERVs decay by neutral evolution; however, many ERV fragments have been co-opted by the host to function as protein-coding genes or regulatory elements (Johnson, 2019; Thompson *et al.*, 2016; Wang and Han, 2020). Thus, retroviruses are important contributors to disease and evolution.

Retroviruses encode three main genes, in the following order: 5′-gag-pol-env-3′. Each gene produces several proteins, including viral structural proteins, a protease, reverse transcriptase and viral envelope proteins. Some retroviruses also encode small ‘accessory’ proteins near the 3′ end. In short, retroviruses have a largely consistent genome organization.

We report that some primate ERVs encode an extra protein upstream of the gag gene (or possibly fused to the gag gene). This protein is homologous to protein MC132 of the human poxvirus Molluscum contagiosum. MC132 suppresses the immune system by targeting NF-κB, and it had no known homologs until now (Brady *et al.*, 2015).

## 2 Results

### 2.1 MC132 protein fossils in human ERVs

**Fig. 1.**
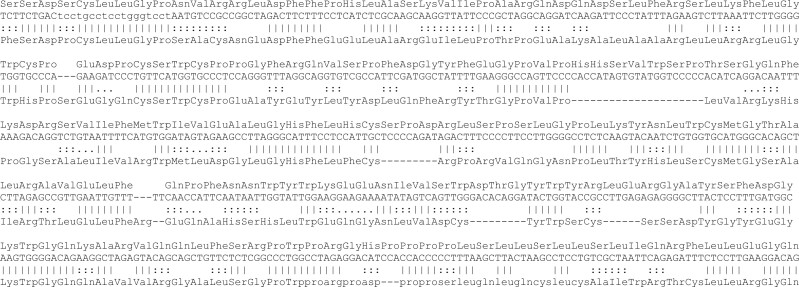
Alignment between Dfam’s HERV30 consensus DNA sequence and MC132 protein (Q98298). The DNA’s translation is shown above it. ‖| indicates a match, ::: a positive substitution score, and … a zero substitution score (Fig. 7). The alignment has 36% identity. Lowercase regions were deemed to be simple repeats by the alignment tool (LAST)

We discovered this ERV protein by chance, while searching for protein fossils in the human genome. Protein fossils are segments of formerly protein-coding DNA, and we recently developed a sensitive method to find them by comparing DNA to protein sequences to find homologous segments (Frith, 2022; Yao and Frith, 2022). We found a few dozen segments of the human genome with homology to protein MC132 from Molluscum contagiosum virus (specifically, proteins Q98298 and A0A1S7DLX6 from UniProt release 2022_03). These segments lie in ERVs. There are many types of human ERV, and these segments lie in a few specific types. We used ERV annotation from RepeatMasker, which finds ERVs by comparing the genome to ERV models in the Dfam database (Storer *et al.*, 2021). These models and annotation are not perfect (see below), but according to them, the protein has homology to HERV30 (Fig. 1), HERV17, HERV9 and perhaps a few others. These ERV types are closely related: they are in Dfam’s ERV1 class.

### 2.2 Reconstructing the ancestral sequence

These ERVs presumably inserted into the genome millions of years ago and underwent random mutations, degrading the original sequence. We attempted to reconstruct an ancestral sequence, from 15 DNA segments whose alignments to MC132 cover most of the protein (<10 amino acids missing from the start and <30 missing from the end). We fed these segments, plus 200 bp flanks, to Refiner (Hubley *et al.*, 2022). Refiner inferred an ancestral DNA sequence, which remarkably has an 879-bp open reading frame (ORF) encompassing the MC132 homology. This ORF encodes the following protein:

MAPPEAPPAXVTERETATSSDPCLLGPNVRRLDFFPHLAS

KVIPARQDQDSLFRSLKFLGWRPEDPCSWCPPGFRQVSPF

DGYFEGPVPHHSVWSPTSGQFKDRSVIFMWIVEALGHFLH

CSPDRLSPSLGPLKYNLWCMGTALRAVELLFQPFNNWYWK

EENIVSWDTGYWYRLERGAYSFDGKWGQKARVQQLFSRPW

PRGHPPPPLSLLSLLSLIQRFLLEGQFYGQAHVNWALACK

HQWCPRPRPCHPGTGRTRWQKDHNKSNSPCAPFSGQWAHG

RGKGSFHPAGKHG

It is easy to verify (e.g. by NCBI BLAST) that this protein has significant similarity to MC132. The DNA from Refiner is 99% identical to Dfam’s HERV30 consensus sequence, but the latter has two frame shifts disrupting the ORF.

**Fig. 2.**
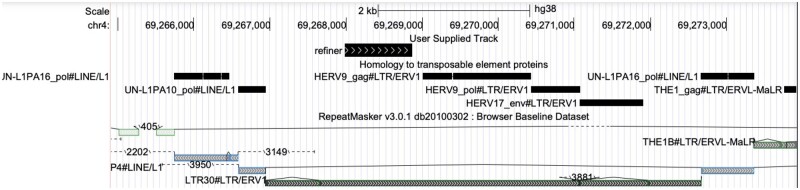
Location of the newly-discovered protein in an ERV in human chromosome 4. The location of the new protein is shown by the top bar labeled ‘refiner’. The black bars below that show DNA segments aligned to known transposable element proteins [from a previous study (Frith, 2022)], which are in the usual gag-pol-env order. Below that are RepeatMasker annotations of transposable element-derived segments. Here, RepeatMasker annotates two long terminal repeats of type LTR30, flanking an internal retroviral sequence of type HERV30. There is a 3881-bp deletion near the end of this internal sequence. Screenshot from the UCSC genome browser (http://genome.ucsc.edu) (Kent *et al.*, 2002)

We then sought human genome segments homologous to this new protein, using the same DNA-versus-protein homology search method as above (see Methods). We found hundreds of hits (Table 1), mostly in HERV17 annotations (Table 2). The hits are consistently upstream of the gag gene (Fig. 2). Remarkably, there is one HERV30 in chromosome 4 where the ORF is intact, with no frame disruptions (Fig. 2).

**Table 1.** Alignments of the reconstructed protein sequence and Q98298 in each genome

Organism	Refiner sequence	Q98298	Refiner + Q98298
Human	252	65	317
Gibbon	221	46	267
Rhesus	232	61	293
Golden snub-nosed monkey	253	43	296
Marmoset	267	92	359
Bushbaby	7	4	11

**Table 2.** Overlaps between the protein matches and RepeatMasker ERV annotations

Organism	HERV17	HERV9	HERV9N	HERV30	HERVIP10FH	HERVK14	Other
Human	156	45	39	23	8	2	33
Gibbon	120	45	33	16	5	3	9
Rhesus	155	100	0	18	5	2	4
Golden snub-nosed monkey	151	100	0	24	7	2	4

### 2.3 The protein homology overlaps gaps in HERV17 annotations

**Fig. 3.**
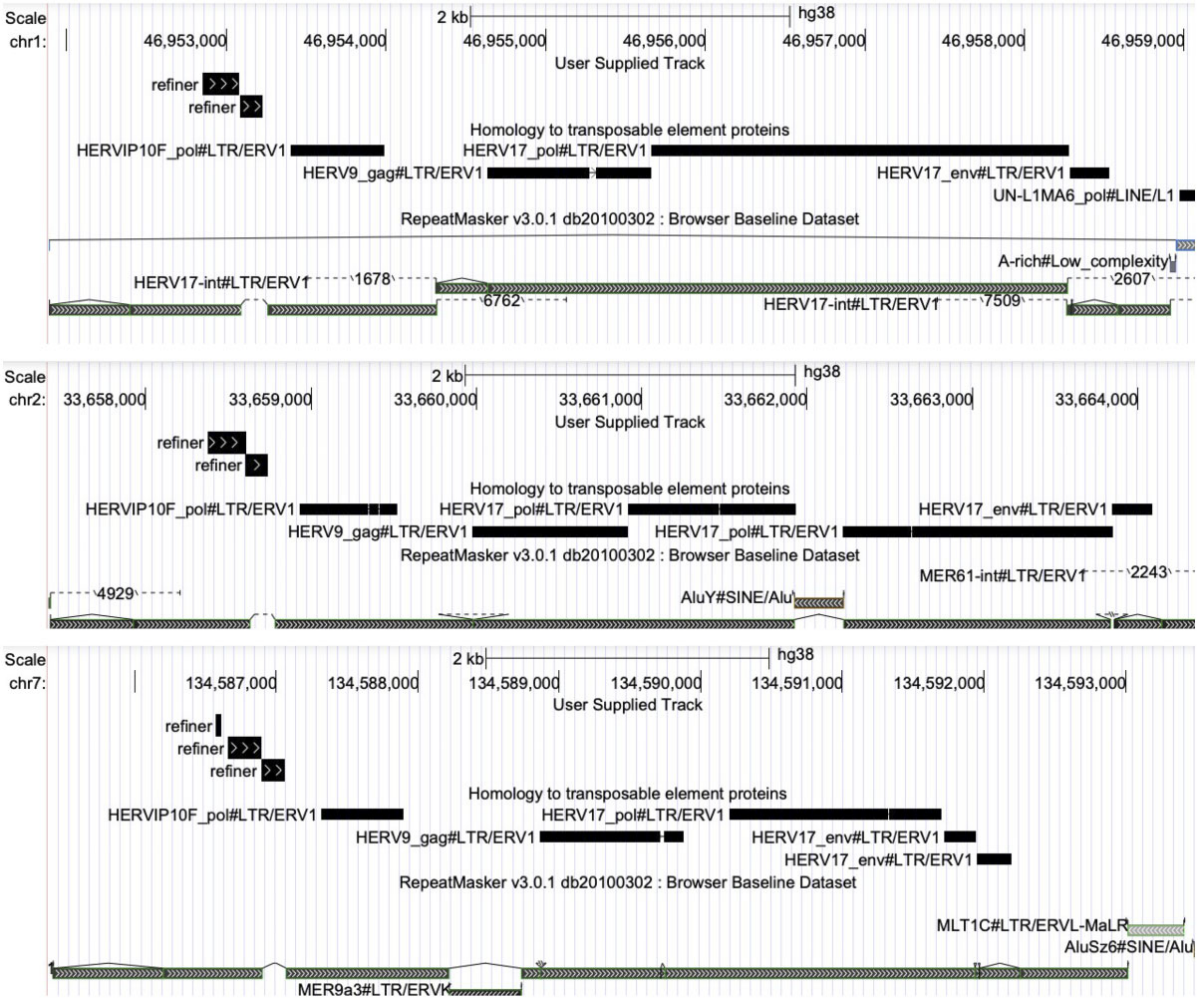
The new protein overlaps gaps in HERV17 annotations. The panels show three human genome locations with homology to the new protein (bars labeled ‘refiner’). The protein aligns to each location as two or three separate fragments. The black bars below that show DNA segments aligned to known transposable element proteins. Below that are RepeatMasker annotations of transposable element-derived segments. In each case, the new protein overlaps a retroviral sequence of type HERV17. However, in each case, the protein overlaps a consistent gap in RepeatMasker’s HERV17 annotation. There is also an unexpected pol protein homology (HERVIP10F_pol) between the new protein and the gag gene

We noticed that, in HERV17, the DNA region homologous to the new protein overlaps a consistent gap in RepeatMasker’s HERV17 annotation (Fig. 3). This gap indicates an imperfection in the ERV models used by RepeatMasker. Either the HERV17 model is inaccurate in this region or we have a new HERV17-like subfamily that is not yet represented in RepeatMasker’s models. We made a new model, by feeding HERV17 sequences from the human genome to Refiner. The new consensus sequence is 99% identical to Dfam’s HERV17 over most of its length but has an extra 270 bp in the gap region. This suggests that we should update the model rather than add a new subfamily. On the other hand, a previous study suggested two HERV17 subgroups (Grandi *et al.*, 2016): the extra 270 bp was not discussed but is actually present in one subgroup. In any case, the gap region is immediately upstream of a GA tandem repeat, which may evolve quickly and cause variation in these models.

Our new consensus sequence has frameshifts in the region homologous to the new protein. So do the previous subgroups. Thus, HERV17 may have proliferated in the genome after disruption of the reading frame. HERV17 (also known as HERV-W) is interesting because it has many copies that were retrotransposed by LINE enzymes (Pavlíček *et al.*, 2002), and its env gene was co-opted as the human syncytin gene *ERVW-1* involved in placental development (Mi *et al.*, 2000).

### 2.4 Extent of protein homology in ERV families

**Fig. 4.**
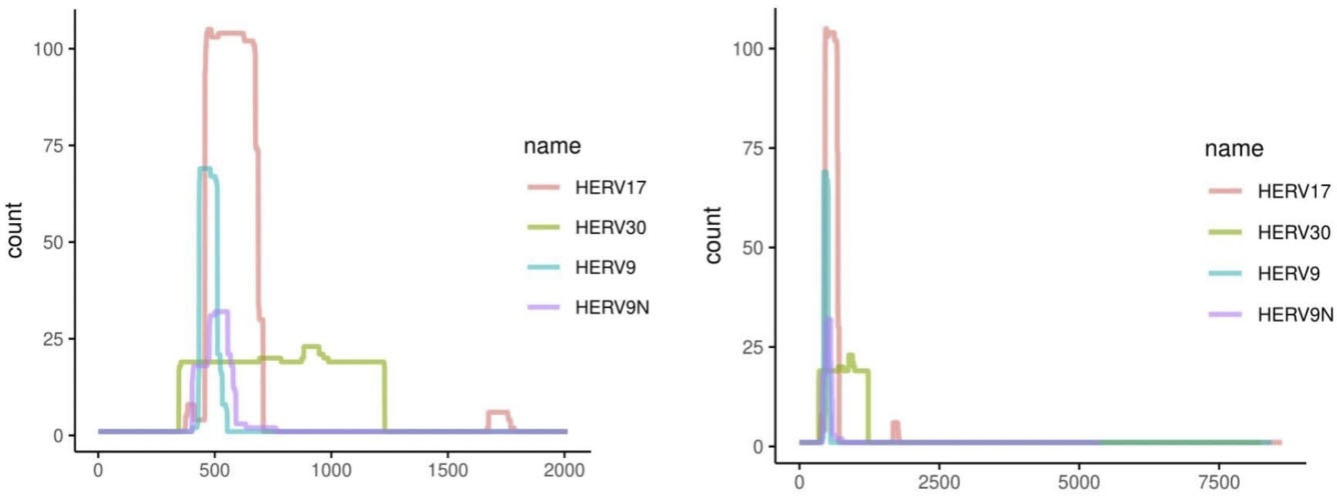
Locations of the newly-found protein in four ERV families. The right-hand panel shows the full-length ERVs, and the left-hand panel is zoomed in to the matching region. HERV30 has a long match to the protein (green), while the other ERVs have fragmentary matches. The separated matches in HERV17 (red) appear to correspond to the annotation gap shown in Figure 3

To better understand this protein homology in each ERV family, we took the genome segments aligned to the Refiner protein and mapped them to Dfam’s consensus DNA sequence for each ERV (Fig. 4). HERV17 consistently has a partial match, shorter than the HERV30 match, while HERV9 and HERV9N have even shorter matches.

In some of these cases, the RepeatMasker ERV annotations are fragmented and suggest ambiguity about which type of ERV1 is really present. It is possible that some of the Dfam consensus sequences incorrectly combine different ERV subfamilies or that some ERVs are actually chimeric. Careful reconstruction of these ERVs would help us to understand the evolution of this protein.

### 2.5 The new ERV protein in non-human primates

Finally, we searched several mammal genomes (Table 3) for homologies to MC132 and the new protein from Refiner. Similarly to human, there are hundreds of hits in apes, old-world monkeys and new-world monkeys (Table 1). Among other primates, there are a few hits in bushbaby, but none in tarsier or mouse lemur. This is surprising, because it is usually thought that bushbaby and lemurs are related as Strepsirrhini, whereas tarsiers and simians are related as Haplorhini. We found no hits in other mammals (e.g. rat, pika, dolphin).

**Table 3.** Genome versions

Organism	Species	Genome assembly	RepeatMasker version
Human	*Homo sapiens*	GRCh38.p14	4.1.0
Gibbon	*Nomascus leucogenys*	Asia_NLE_v1	4.0.8
Rhesus	*Macaca mulatta*	Mmul_10	4.0.8
Golden snub-nosed monkey	*Rhinopithecus roxellana*	Novogene Rrox_v1	4.0.8
Marmoset	*Callithrix jacchus*	Callithrix_jacchus_cj1700_1.1	4.0.8
Tarsier	*Tarsius syrichta*	tarSyr2	4.0.5
Mouse lemur	*Microcebus murinus*	Mmur_3.0	4.0.6
Bushbaby	*Otolemur garnettii*	OtoGar3	4.0.6
Norway rat	*Rattus norvegicus*	mRatBN7.2	4.0.8
Pika	*Ochotona princeps*	OcjPri4.0	4.0.8
Dolphin	*Tursiops truncatus*	mTurTru1.mat.Y	4.0.8

**Fig. 5.**
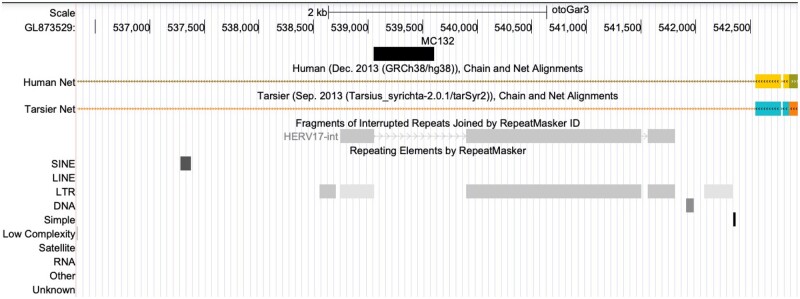
The new protein matches a gap in ERV annotation in the bushbaby genome. The black bar shows a genome segment homologous to MC132, and the gray bars show RepeatMasker annotations. The colored bars show alignments to human and tarsier genomes: these alignments do not cover the segment homologous to MC132

As expected, the homologous segments of these primate genomes lie in ERVs. In apes and old-world monkeys, these are the same types of ERV as in human, according to RepeatMasker annotations. In a more distantly related new-world monkey (marmoset), we find the highest number of homologous segments, which almost all lie in ERV annotations of type ERV1-1_CJa-I (also in the ERV1 category). In bushbaby, the hits overlap gaps in ERV annotations (Fig. 5). It is likely that the ERV models used by RepeatMasker become less accurate for primates more distantly related to human.

**Fig. 6.**
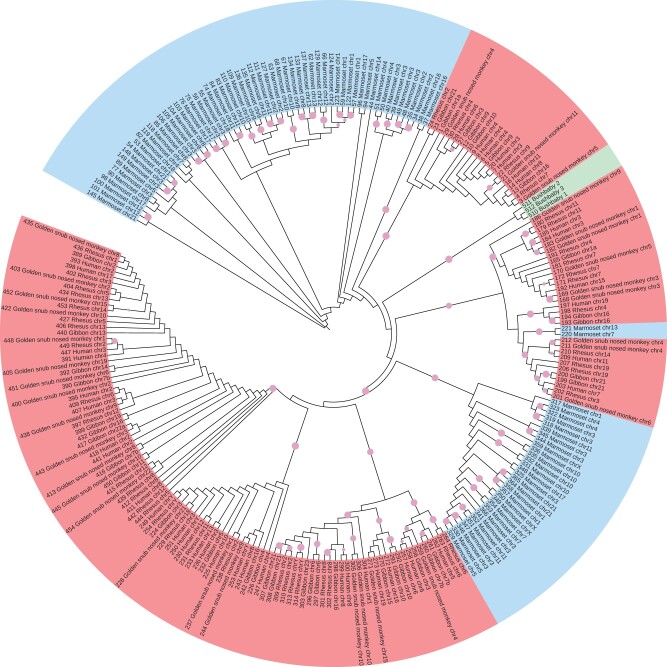
Evolutionary tree of protein fossils homologous to MC132. Blue indicates fossils from Platyrrhini (new-world monkey) genomes, red Catarrhini (apes and old-world monkeys), and green bushbaby. Pink circles mark branches with medium-to-high confidence (bootstrap value >70%)

We tried to infer the evolutionary tree of DNA segments homologous to the new protein (Fig. 6). The tree largely groups the DNA segments according to their three main primate clades: Catarrhini (apes and old-world monkeys), new-world monkeys and bushbaby. On the other hand, the tree does not separate apes (e.g. human) from old-world monkeys (e.g. rhesus). This indicates that these ERVs proliferated in the three main clades more recently than their last common ancestors, and in Catarrhini before the last common ancestor of apes and old-world monkeys.

## 3 Discussion

Some primate retroviruses used to encode an additional protein, homologous to an immune-suppressing protein in a human poxvirus. It is plausible that this retrovirus protein also had an immune-suppressing function. Perhaps some extant retroviruses still encode such a protein. We found one intact ORF for this protein in human chromosome 4, so it is possible that this protein is present in humans. The ORF might have been co-opted as a gene that (down-)regulates immune responses.

One puzzle is that the ORF upstream of gag in a retrovirus would be expected to hinder the translation of gag. So, it is possible that the ORF’s stop codon inferred by Refiner is incorrect, and it was actually one long ORF fused to gag.

Since retroviruses and poxviruses are not closely related, DNA encoding this protein must have been horizontally transferred between these types of virus. The direction of transfer is unknown and could be indirect, e.g. from an unknown third source. In any case, a retrovirus encoding this protein infected ancient primates. The inferred evolutionary tree (Fig. 6) suggests that there were independent infections of Catarrhini, new-world monkeys and bushbaby ancestors. Homologs of this protein may lurk elsewhere, and finding them should clarify its evolutionary history.

## 4 Methods

### 4.1 DNA-versus-protein homology search

DNA-versus-protein homology searches were done with LAST version 1411, essentially as described previously (Frith, 2022):

lastdb -q -c myDB proteins.fasta

lastal -D1e9 -K1 -p my.train myDB genome.fasta > out.maf

**Fig. 7.**
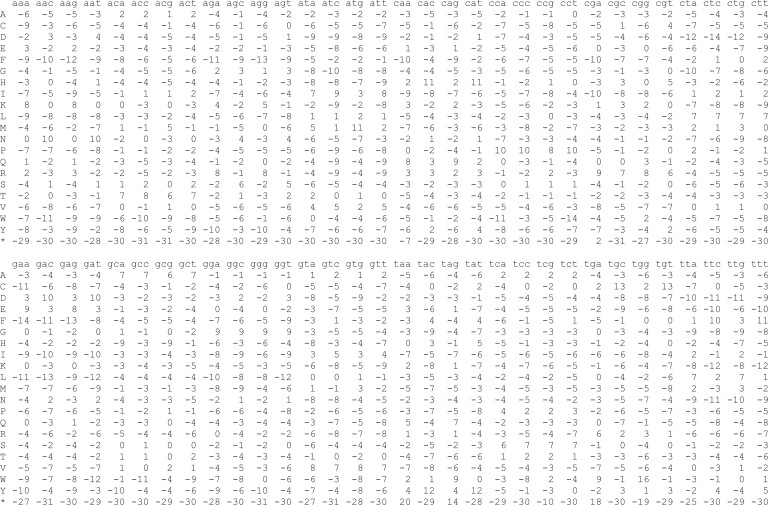
The substitution score matrix inferred by last-train

This requires a file ‘my.train’ specifying rates of substitution, deletion, and insertion. These rates can be inferred by finding homologies between DNA and protein sequences using last-train (Frith, 2022; Yao and Frith, 2022). However, it is not obvious which sequences to use for this inference: they must have extensive-enough homology to infer the parameters of a 64 × 21 substitution matrix (Fig. 7). We used human pseudogene DNA and non-human proteins: the idea is that they have diverged by a combination of protein-coding and noncoding evolution. Specifically, we used retrogene DNA in human genome hg38 according to ucscRetroInfo9 (Baertsch *et al.*, 2008) and chicken proteins from UniProt release 2022_03 (proteome UP000000539) (The UniProt Consortium, 2021). Instead of chicken, we also tried mouse and zebrafish, but it did not seem to make much difference. The rates were inferred like this:

lastdb -q -c db UP000000539_9031.fasta

last-train ––codon ––pid=50 -m100 db retro.fasta > my.train

### 4.2 DNA consensus sequences

Refiner, from RepeatModeler version 2.0.3, was run with default parameters.

### 4.3 Evolutionary tree inference

We aligned DNA segments >300 bp and then inferred their evolutionary tree, using MAFFT version 7 (Katoh *et al.*, 2019) with these options: ––add, ––keeplength, MaxAlign v1.1 (Gouveia-Oliveira *et al.*, 2007), and neighbor-joining with the Jukes-Cantor model and 1000 bootstrap replicates. We displayed the tree with iTOL v6 (Letunic and Bork, 2021).

## Data Availability

The data underlying this article are available in the article.
